# Review and evaluation of performance measures for survival prediction models in external validation settings

**DOI:** 10.1186/s12874-017-0336-2

**Published:** 2017-04-18

**Authors:** M. Shafiqur Rahman, Gareth Ambler, Babak Choodari-Oskooei, Rumana Z. Omar

**Affiliations:** 1 Institute of Statistical Research and Training, University of Dhaka, Dhaka, Bangladesh; 2 Department of Statistical Science, University College London, London, UK; 3 Institute of Clinical Trials & Methodology, University College London, London, UK

**Keywords:** Prognostic model, discrimination, calibration, Validation, Survival analysis

## Abstract

**Background:**

When developing a prediction model for survival data it is essential to validate its performance in external validation settings using appropriate performance measures. Although a number of such measures have been proposed, there is only limited guidance regarding their use in the context of model validation. This paper reviewed and evaluated a wide range of performance measures to provide some guidelines for their use in practice.

**Methods:**

An extensive simulation study based on two clinical datasets was conducted to investigate the performance of the measures in external validation settings. Measures were selected from categories that assess the overall performance, discrimination and calibration of a survival prediction model. Some of these have been modified to allow their use with validation data, and a case study is provided to describe how these measures can be estimated in practice. The measures were evaluated with respect to their robustness to censoring and ease of interpretation. All measures are implemented, or are straightforward to implement, in statistical software.

**Results:**

Most of the performance measures were reasonably robust to moderate levels of censoring. One exception was Harrell’s concordance measure which tended to increase as censoring increased.

**Conclusions:**

We recommend that Uno’s concordance measure is used to quantify concordance when there are moderate levels of censoring. Alternatively, Gönen and Heller’s measure could be considered, especially if censoring is very high, but we suggest that the prediction model is re-calibrated first. We also recommend that Royston’s D is routinely reported to assess discrimination since it has an appealing interpretation. The calibration slope is useful for both internal and external validation settings and recommended to report routinely. Our recommendation would be to use any of the predictive accuracy measures and provide the corresponding predictive accuracy curves. In addition, we recommend to investigate the characteristics of the validation data such as the level of censoring and the distribution of the prognostic index derived in the validation setting before choosing the performance measures.

## Background

Prediction models are often used in the field of healthcare to estimate the risk of developing a particular health outcome. These prediction models have an important role in guiding the clinical management of patients and monitoring the performance of health institutions [1, 2]. For example, models have been developed to predict the risk of in-hospital mortality following heart valve surgery and to predict the risk of developing cardiovascular disease within the next 10 years [3, 4]. Given their important role in health research, it is essential that the performance of a prediction model is evaluated in data not used for model development, using appropriate statistical methods [5, 6]. This model evaluation process is generally termed ‘model validation’ [7, 8]. The general idea of validating a prediction model is to establish that it performs well for new patients. Different types of validation process have been discussed in the literature [5–8]. The most commonly used processes include (i) splitting a single dataset (randomly or based on time) into two parts, one of which is used to develop the model and the other used for validation, (internal or temporal validation) and (ii) validating the model using a new dataset collected from a relevant patient population in different centres (external validation). Of the two approaches, external validation investigates whether a prediction model is transportable (or generalisable) to new patients.

When validating a prediction model, the predictive performance of the model is commonly addressed by quantifying: (i) the ‘distance’ between the observed and predicted outcomes (overall performance); (ii) the ability of the model to distinguish between low and high risk patients (discrimination); (iii) the agreement between the observed and predicted outcomes (calibration) [8]. Performance measures based on these concepts are well established for risk models for binary outcomes [1, 9, 10], but that is not the case for risk models for survival outcomes (survival prediction models) where censoring complicates the validation process [6].

Several performance measures have been suggested for use with survival prediction models. However, a few of these are not appropriate for use with validation data without modification. Also, some require specification of a clinically appropriate time-point or region to match the aims of the validation study. Some of these performance measures have been reviewed previously [11–17], although only two of the reviews were in the context of model validation. These were Hielscher et al. who reviewed ‘overall performance’ measures, and Schmid and Potapov who reviewed discrimination measures [12, 16]. Consequently, it is still unclear which performance measures should be routinely used in practice when validating survival prediction models using external data.

A good performance measure should be unbiased in the presence of censoring in the validation data. If this were not the case, the level of censoring would affect the evaluation of model performance and a high level of censoring might lead to an over-optimistic verdict regarding the performance of the prediction model. In addition, a good measure should be straightforward to interpret and, ideally, should be easy to implement or available in widely used software.

The aim of this paper is to review all types of performance measures (overall performance, discrimination and calibration) in the context of model validation and to evaluate their performance in simulation datasets with different levels of censoring and case-mix. Where necessary, measures have been modified to allow their use with validation data and a case study is provided to describe how these measures can be estimated in practice. Recommendations are then made regarding the use of these measures in model validation.

## Methods

### Data

Two datasets, which have previously been used to develop clinical prediction models, were used as the basis of the simulation study. They differ with respect to event rates, level of censoring, types of predictors and amount of prognostic information.

### Breast cancer data

This dataset contains information on 686 patients diagnosed with primary node positive breast cancer from the German Breast Cancer Study [18]. The outcome of interest is recurrence-free survival time and the dataset contains 299 (44%) events. The median follow-up time is 3 years. The predictors are age, number of positive lymph nodes, progesterone receptor concentration, tumour grade (1–3), and hormone therapy (yes/no). These data have been analysed previously by Sauerbrei and Royston and their Model III was used as the basis for simulation [19]. That is, the continuous predictors were all transformed using fractional polynomials (FPs) and tumour grade was dichotomised (1/2–3). Number of positive lymph nodes and progesterone receptor concentration were each modelled using one FP term whereas age was modelled using two FP terms.

### Hypertrophic cardiomyopathy data

This dataset contains information on a retrospective cohort of 1504 patients with hypertrophic cardiomyopathy (HCM) from a single UK cardiac centre [20]. The outcome of interest is sudden cardiac death or an equivalent event, i.e., a composite outcome and the dataset contains just 84 (6%) events. The median follow-up time is over 6 years. The predictors of interest are age, maximal wall thickness, left atrial diameter, left ventricular outflow gradient, family history of sudden cardiac death (yes/no), non-sustained ventricular tachycardia and unexplained syncope (yes/no). The prediction model produced by O’Mahony et al. was used as the basis for simulation [20]. In particular, maximal wall thickness was modelled using linear and quadratic terms.

### Prediction models for survival outcomes

Prediction models for survival outcomes are commonly developed using the Cox proportional hazards model and hence this model was used in the simulations [5, 21]. The Cox model

$$ \mathrm{h}\left(\mathrm{t}\Big|\mathbf{x}\right)={\mathrm{h}}_0\left(\mathrm{t}\right) \exp \left(\upeta \right) $$

models the hazard *h*(*t*|***x***) at time t as a product of a nonparametric baseline hazard *h*_0_(*t*) and the exponential of the prognostic index *η* = *β*_1_ *x*_1_ + … + *β*_*p*_ *x*_*p*_ = ***β*** ^*T*^ ***x***. The latter is a linear combination of *p* predictor values with regression coefficients *β*_1_, …, *β*_*p*_ providing weights. The predictive form of this model can be written in terms of the survival function as

$$ S\left( t\Big|\boldsymbol{x}\right)={S}_0{(t)}^{\exp \left(\eta \right)} $$

where *S*(*t*|***x***) is the probability of surviving beyond time *t* given predictors ***x***, and *S*_0_(*t*) is the baseline survivall function at time t, where *S*_0_(*t*) = exp[−∫_0_^*t*^ *h*_0_(*u*)*du*]. To make predictions at a specific time-point *τ*, one requires estimates $\widehat{\boldsymbol{\beta}}$ and ${\hat{S}}_0\left(\tau \right)$.

### Performance measures for survival prediction models

Measures were selected for investigation on the basis of their performance in previous reviews [11–16], their ease of interpretation, and their availability, or ease of implementation, in statistical software. The selected performance measures are now described in the context of model validation. All measures were implemented in Stata using either built-in or user-written routines [22].

### Measures of overall performance

Six measures of ‘overall performance’ were selected, of which four are based on predictive accuracy and two on explained variation [14]. These ‘R^2^-type’ measures typically take values between 0 and 1, though negative values may be possible in validation data if the prediction model is out-performed by the null model that has no predictors. This issue is discussed later.

The measures based on predictive accuracy were Graf et al’s R^2^ measure and its integrated counterpart [23], Schemper and Henderson’s R^2^ measure [24], and a modified version of the latter based on Schmid et al. [25]. The measures based on explained variation were Kent and O’Quigley’s R^2^_PM_ [26], and Royston and Sauerbrei’s R^2^ version of their separation statistic D [27]. Nagelkerke’s R^2^ measure was not considered due to its known poor performance in the presence of censoring [26, 28].

### Graf et al’s R^2^_BS_ and R^2^_IBS_

The R^2^_BS_ measure proposed by Graf et al. is based on quantifying prediction error at a time-point *τ* using a quadratic loss function [21]. Specifically, *R*_*BS*_^2^(*τ*) = 1 − *BS*(*τ*|*X*)/*BS*(*τ*) where

$$ B S\left(\tau \Big| X\right)={\displaystyle {\int}_X E}\left[{\left( I\left( T>\tau \right)-\hat{S}\left(\tau \Big| X\right)\right)}^2\right] d{F}_X(X) $$

is the prediction error at time *τ* for the prediction model and I(T > *τ*) is the individual survival status at this time-point. Similarly, BS(τ) is the prediction error for the null model at the same time-point, and is based on the survival function $\hat{\mathrm{S}}\left(\uptau \right)$ from the null model. The integrated version, R_IBS_^2^(τ), is defined in a similar way to R_BS_^2^(τ) but involves integrating both BS(t|x) and BS(t) over the range [0, *τ*].

The calculation of prediction errors for both of these models in validation data requires estimates of the corresponding baseline survival functions. This, however, is rarely provided by model developers [6]. One solution might be to estimate these survival functions by re-fitting the Cox model with the PI as the sole predictor in the validation data. This is the approach that we took when calculating R^2^_BS_ and R^2^_IBS_, and the R^2^_SH_ and R^2^_S_ measures described below.

### Schemper and Henderson’s R^2^_SH_ and Schmid et al’s R^2^_S_

The R^2^ measure proposed by Schemper and Henderson (denoted here by R^2^_SH_) is similar to Graf et al’s R^2^_IBS_ but is based on an absolute loss function [24]. This loss function was chosen to reduce the impact of poorly predicted survival probabilities, which are likely to occur in the right tail of the survival distribution. Specifically, *R*_*SH*_^2^(*τ*) = 1 − *D*(*τ*|*x*)/*D*(*τ*), where

$$ D\left(\tau \Big| x\right)=2{\displaystyle {\int}_0^{\tau} E}\left[ S\left( t\Big| X\right)\left(1- S\left( t\Big| X\right)\right)\right] f(t) d t W\left(\tau \right) $$

is the prediction error at time τ for the prediction model and *W*(*τ*) = 1/∫_0_^*τ*^ *f*(*t*)*dt* is a weight function to compensate for the measure being defined only on (0, τ). Similarly, D(τ) is the prediction error for the null model.

Schmid et al. prove that Schemper and Henderson’s estimator of D(τ|x) and D(τ) is not robust to model misspecification and suggest an improved estimator [25]. We estimated a summary measure, denoted by R^2^_S_, based on this estimator.

### Kent and O’Quigley’s R^2^_PM_

Kent and O’Quiqley’s proposed their R^2^_PM_ measure for the Cox model based on the definition of R^2^ for linear regression [26]. That is,

$$ {R}_{PM}^2=\frac{Var\left(\eta \right)}{Var\left(\eta \right)+{\sigma}_{\epsilon}^2} $$

seeks to quantify the proportion of variation in the outcome explained by the predictors in the prediction model, where *σ*_*ϵ*_^2^ ≅ *π* ^2^/6 is the variance of the error term in an equivalent Weibuill model [13].

This measure does not use the observed survival times directly in its calculation and instead relies on the prediction model being correctly specified. As a result, R^2^_PM_ could be misleading if an apparent strong relationship between the outcomes and predictors in development data is not reproduced in validation data. To overcome this deficiency, we suggest re-calibrating the prediction model to the validation dataset before calculation of R^2^_PM_. This procedure will tend to reduce the value of R^2^_PM_ and is described later.

### Royston and Sauerbrei’s R^2^_D_

Royston and Sauerbrei’s R^2^_D_ is similar to R^2^_PM_ but is based on the authors’ own D statistic, a measure of prognostic separation described later. That is,

$$ {R}_D^2=\frac{D^2/{\kappa}^2}{D^2/{\kappa}^2+{\sigma}_{\epsilon}^2} $$,

where $\kappa =\sqrt{8/\pi}$ [27]. The ratio *D* ^2^/*κ* ^2^ is an estimator of Var(η), provided that η is Normally distributed.

### Measures of discrimination

Four measures of discrimination were selected, of which three are based on concordance and one on prognostic separation. Discrimination measures assess how well a model can distinguish between low- and high- risk patients, and concordance measures in particular quantify the rank correlation between the predicted risk and the observed survival times. Concordance measures usually take values between 0.5 and 1, where a value of 0.5 indicates no discrimination and a value of 1 indicates perfect discrimination. The selected concordance measures were those of Harrell [29], Uno et al. [30], and Gönen and Heller [31], and the selected prognostic separation measure was Royston and Sauerbrei’s D statistic [27].

### Harrell’s C_H_

The concordance probability is the probability that of a randomly selected pair of patients (i,j), the patient with the shorter survival time has the higher predicted risk. Formally,

$$ C= P\left({\eta}_i>{\eta}_j\Big|{T}_i<{T}_j\right) $$

where η_i_ and η_j_ are the prognostic indices for patients i and j, and T_i_ and T_j_ are the corresponding survival times. Harrell’s estimator C_H_ considers all usable pairs of patients for which shorter time corresponds to an event and estimates C_H_ as the proportion of these pairs for which the patient with the shorter survival time has the higher predicted risk [31]. A modified version of this estimator, C_H_(τ), restricts the calculation to include just those patient pairs where *T*_*i*_ < *τ* and may provide more stable estimates [29, 30]. This truncated version may also be preferred if one were primarily interested in the discrimination of a prediction model over a specified period, for example within 5 years [20].

### Uno et al’s C_U_

In the presence of censoring C_H_ and C_H_(τ) are biased, even under independent censoring, as they ignore patient pairs where the shorter observed time is censored [15, 32]. Due to this deficiency, Uno et al. [30] proposed a modified estimator C_U_(τ) that uses weightings based on the probability of being censored. Furthermore, like C_H_(τ), the calculation may also be restricted to include just those patient pairs where *T*_*i*_ < *τ*. Uno et al. found that their estimator was reasonably robust to the choice of τ, but noted that the standard error of the estimate could be quite large if τ were chosen such that there was little follow-up or few events beyond this time point [29].

### Gonen and Heller’s C_GH_

Gönen and Heller proposed an alternative estimator C_GH_ based on a reversed definition of concordance [30],

$$ K= P\left({T}_i<{T}_j\Big|{\eta}_i>{\eta}_j\right), $$

which is the probability that of a randomly selected pair of patients (i, j), the patient with the higher predicted risk has the shorter survival time. To avoid bias caused by censoring, their estimator is a function of the model parameters and the predictor distribution and assumes that the proportional hazards assumption holds.

As with R^2^_PM_, C_GH_ does not use the observed event and censoring times in its calculation and relies on the prediction model being correctly specified [15]. Therefore, we suggest re-calibrating the prediction model to the validation dataset before calculating C_GH_.

### Royston and Sauerbrei’s D

The D statistic is a discrimination measure that quantifies the observed separation between subjects with low and high predicted risk [27]. Specifically, D estimates *κσ*, where *σ* is the standard deviation of the prognostic index and $\kappa =\sqrt{8/\pi}$. The scale factor *κ* enables D to be interpreted as the log hazard ratio that compares two equal-sized risk groups defined by dichotomising the distribution of the patient prognostic indices at the median value.

### Measures of calibration

One calibration measure was selected, the commonly used calibration slope proposed by van Houwelingen [33], which is based on the analogous measure for binary outcomes [34, 35].

### Calibration slope

The calibration slope is simply the slope of the regression of the observed survival outcomes on the predicted prognostic index [33]. It is estimated by fitting a Cox model to new survival outcomes with the predicted prognostic index, $\hat{\eta}$, as the sole predictor in the model

$$ h\left( t\Big| x\right)={h}_0(t) \exp \left({\alpha}_1\hat{\eta}\right). $$

Values of ${\hat{\alpha}}_1$ close to 1 suggest that the prediction model is well calibrated. Moderate departures from 1 indicate that some form of model re-calibration may be necessary. In particular, ${\hat{\alpha}}_1<<1$ suggests over-fitting in the original data with predictions that may be too low for low risk patients or too high for high risk patients.

A brief summary of the performance measures is given in Table 1.

**Table 1:** Summary of the performance measures

Types of Measures	Measures	Characteristics	Range and Interpretation	Software
Overall Performance	R^2^_BS_	Assesses relative gain in predictive accuracy quantified using at a specific time point based on squared error loss function.	Range: 0 to 1 Interpretation: % gain in predictive accuracy at a single time point relative to the null model.	Available in SAS and R and easy to implement in other software
	R^2^_IBS_	Same approach as R^2^_BS_ but provides a summary over a range of time period.	Range: same as R^2^_BS_ Interpretation: % gain in predictive accuracy over a range of time period relative to the null model.	Available in SAS and R and easy to implement in other software
	R^2^_SH_	Assesses relative gain in predictive accuracy quantified based on absolute error loss function. It is not robust to model mis-specification.	Same as R^2^_IBS_	Available in SAS and R and easy to implement in other software
	R^2^_S_	Modified version of R^2^_SH_ which is robust to model mis-specification.	Same as R^2^_IBS_	Available in SAS and R and easy to implement other software
	R^2^_PM_	Measures the variation in the outcome explained by the covariates in the model. Assume that the model is correctly specified. Requires re-calibration in the validation data.	Range: 0 to 1 Interpretation: % of explained variation by the model.	Easy to implement in any software
	R^2^_D_	Measures the relative gain in prognostic separation quantified by the D statistic. Assume that the PI is normally distributed.	Range: 0 to 1 Interpretation: % of prognostic separation explained by the model.	Available in Stata and easy to implement in other software
Discrimination	C_H_	Rank order statistic based on usable pairs in which shorter time corresponds to an event.	Range: 0.5 to 1 Interpretation: probability of correct ordering for a randomly selected pair of subjects.	Available in R and Stata and easy to implement in software
	C_U_	Rank order statistic based on usable pairs. Inverse probability weighting is used to compensate for censoring.	Same as C_H._	Available in R and easy to implement in other software
	C_GH_	Rank order statistic based on all patient pairs. Assumes that Cox PH model is correctly specified.Requires re-calibration in the validation data.	Same as C_H._	Available in R and Stata and easy to implement in other software
	D	Quantifies the observed separation between low and high risk groups. Assumes that PI is normally distributed.	Range: 0 to ∞ Interpretation: log hazard ratio between two equal sized prognostic groups fromed by dichotomising the PI at its median..	Available in Stata and easy to implement in other software
Calibration	Cal Slope	Regression slope of the PI and assesses the agreement between the observed and predicted survival..	Range: −∞ to ∞ Interpretation: a value of 1 suggests perfect calibration and a value much lower than 1 suggest overfitting.	Easy to implement in any software

## Results

### Case study to illustrate the performance measures

A case study is now presented using the breast cancer data in order to describe how the performance measures may be evaluated in a validation setting. The dataset was split randomly into two parts with two thirds of the data used for model development and one third used for model validation. A Cox model was fitted to the development data using the same predictors as in Sauerbrei and Royston’s Model III [19] and the predicted prognostic index was calculated for all patients in the validation data using the estimated regression coefficients $\hat{\upbeta}$. The values of all performance measures are shown in Table 2 with 95% confidence intervals estimated using the bootstrap techniques based on 200 bootstrap samples. For those measures that require specification of a time-point τ, 3 years was deemed to be clinically appropriate. This was also the median follow-up time.

**Table 2:** Values of the performance measures estimated in the breast cancer validation data

Measure	Value (95% CI)
R^2^_IBS_(3)	0.107 (0.036 to 0.178)
R^2^_SH_ (3)	0.130 (0.089 to 0.171)
R^2^_S_ (3)	0.128 (0.090 to 0.167)
R^2^_BS_(3)	0.141 (0.033 to 0.250)
R^2^_PM_	0.194 (0.094 to 0.294)
R^2^_D_	0.192 (0.093 to 0.291)
C_H_	0.674 (0.622 to 0.726)
C_U_	0.666 (0.610 to 0.722)
C_GH_	0.659 (0.616 to 0.701)
C_H_(3)	0.685 (0.633 to 0.737)
C_U_(3)	0.676 (0.619 to 0.734)
D	0.998 (0.672 to 1.323)
Cal. Slope	0.764 (0.531 to 0.996)

**Fig. 1:**
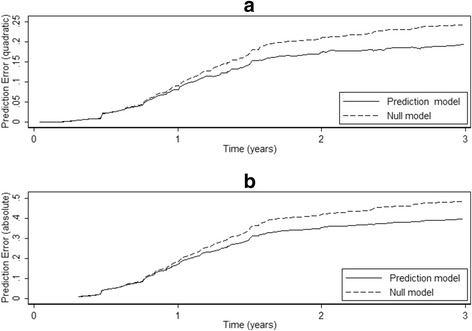
Prediction errors over time for the breast cancer risk model for: **a**) prediction error (based on a quadratic loss function) for calculating R^2^_IBS_ and R^2^_BS_; **b**) prediction error (based on an absolute loss function) for calculating R^2^_SH_

The estimated prediction errors used to estimate R^2^_BS_ and R^2^_IBS_ are shown in Fig. 1a. The errors for the prediction and null models are similar for the first 12 months after which the superiority of the prediction model is evident. The corresponding prediction errors used to estimate R^2^_SH_ appear similar in shape although the magnitude of the errors are larger due to the use of an absolute loss function (Fig. 1b). The prediction errors used to estimate R^2^_S_ are almost indistinguishable from those used to estimate R^2^_SH_ (results not shown). R^2^_IBS_, R^2^_SH_ and R^2^_S_ were estimated after averaging the prediction errors over the first 3 years. As expected R^2^_SH_ and R^2^_S_ are very similar, and both are slightly larger than R^2^_IBS_. R^2^_BS_ was estimated using just the prediction errors at 3 years in Fig. 1a. Its value is larger than that of R^2^_IBS_ as the separation between the prediction errors is close to its maximum at this time-point.

To estimate R^2^_PM_ in the valids first re-calibrated for reasons explained earlier. That is, the Cox model $\mathrm{h}\left(\mathrm{t}\Big|\hat{\upeta}\right)={\mathrm{h}}_0\left(\mathrm{t}\right) \exp \left(\upalpha \hat{\upeta}\right)$ was fitted to the validation data, where $\hat{\upeta}$ is the predicted prognostic index calculated using the regression coefficients estimated in the development data. R^2^_PM_ was then estimated using ${\hat{\upalpha}}^2\mathrm{V}\mathrm{a}\mathrm{r}\left(\hat{\upeta}\right)$ rather than $\mathrm{V}\mathrm{a}\mathrm{r}\left(\hat{\upeta}\right)$. No such re-calibration is required to estimate R^2^_D_ since, unlike R^2^_PM_, it uses the observed survival outcomes in the validation data. The values of R^2^_PM_ and R^2^_D_ are very similar and noticeably larger than the measures based on predictive accuracy (Table 2). We note that a naïve calculation (without re-calibration) of R^2^_PM_ would have produced a much larger value of 0.292, which would have provided an over-optimistic quantification of the model’s predictive performance.

The concordance measures C_H_ and C_U_ were estimated using all usable pairs in which shorter time corresponds to an event, and C_GH_ was estimated after re-calibrating the prediction model to the validation data as described above. The values of these 3 measures are all reasonably similar and would lead to similar conclusions in practice (Table 2). A naïve estimation of C_GH_ (without re-calibration) would have produced a much larger value of 0.696. Restricting the estimation of C_H_ and C_U_ by censoring survival times in the validation data at 3 years produces slightly higher values for both measures, suggesting that the risk model has slightly better discrimination when considering survival over just the first 3 years. The D statistic suggests that the prediction model provides a reasonably high amount of prognostic separation (Table 2). Specifically, if one were to form two risk groups of equal size in the validation data, then the corresponding hazard ratio would be exp(0.998) = 2.71. The calibration slope estimate of 0.76 (equal to the $\hat{\upalpha}$ estimated during the re-calibration process above) suggests that the prediction model has been slightly over-fitted. We note that, in practice, one can detect and adjust for model over-fitting during model development.

The selected measures all provide useful information. R^2^_IBS,_ R^2^_SH,_ and R^2^_S,_ provide a summary measure quantifying the improvement in predictive accuracy offered by the prediction model over the null model. The R^2^_BS_ measure is more appropriate if one is interested in predictive accuracy at a specific time-point, which is sometimes the case in practice. The prediction error curves provide additional insight into the performance of the prediction model at different time-points. R^2^_PM_ and R^2^_D_, which both quantify explained variation, produced very similar values though calculation of R^2^_PM_ required a re-calibration of the prediction model. The concordance measures C_H_, C_U_ and C_GH_ produced similar estimates, though calculation of C_GH_ required the prediction model to be re-calibrated. Additionally, if required, the calculation of C_H_ and C_U_ can be restricted which may be appropriate if one wishes to quantify the discrimination of a prediction model before a specified time-point. Finally, the D statistic produces an intuitive quantification of prognostic separation and the calibration slope provides a succinct indication of whether the prediction model is over-fitted or not.

### Evaluation of the performance measures using simulation

Following the case study, a simulation study was performed to investigate how robust the measures are with respect to both censoring and the characteristics of the validation data. The simulation design is now described.

#### Simulation scenarios

The simulation study is based on the breast cancer and HCM datasets described earlier. For both datasets, development and validation datasets were generated by simulating new outcomes based on a true model and combining these with the original predictor values. Models were fitted in the development data and the performance measures estimated in the validation data. Measures which require a choice of time-point τ (including C_H_ and C_U_) used 3 years for the breast cancer data and 5 years for the HCM data. These values were chosen as they are close to the respective median follow-up times in the original datasets and are conventional choices for survival data. In practice, the choice of time-point would be clinically motivated and based on the underlying research question.

The performance measures were investigated over a range of scenarios to mimic real situations. For all simulations, validation data were constructed to have one of three different risk profiles (denoted low, medium, and high). The use of different risk profiles reflect the fact that, in practice, the characteristics of the patients in the development and validation data may differ [36]. In particular, the event rate for patients in the validation data may be higher or lower than that for patients in the development data due to differences in case-mix.

Four levels of random censoring were considered for the validation datasets (0, 20, 50, and 80%) which combined with the risk profiles, results in a total of 12 validation scenarios for each clinical dataset. The development datasets were generated with no censoring. 5,000 pairs of development and validation datasets were generated for each scenario.

#### Generating new survival and censoring times

Survival times were generated using the Weibull distribution as below

$$ {t}_s={\left(\frac{- \log (u)}{ \exp \left(,\eta \right)}\right)}^{\frac{1}{\gamma}} $$

where η and γ are the observed regression prognostic indices and shape parameter respectively (both used here as the proxy of the true values) and *u* is a uniformly distributed random variable on (0, 1). For the breast cancer data, the prognostic indices and shape parameter were obtained by fitting a Weibull proportional hazards model using the same predictors as in Sauerbrei and Royston’s Model III [19]. For the HCM data, the prognostic indices were based on the regression coefficients estimated by O’Mahony et al. [20] and just the shape parameter was estimated using a Weibull model with the prognostic index specified as an offset.

To introduce random censoring, additional Weibull distributed censoring times were simulated using t_c_ = (−log(u)/λ)^1/γ^ where different choices of the scalar λ were used to give different proportions of censoring. A subject was considered to be censored if their censoring time was shorter than their survival time.

#### Generating validation data with different risk profiles

The three different risk profiles were created in the validation data, by first splitting the patients into tertile risk groups based on their true prognostic index η. It is assumed that the first tertile group consists of low-risk patients, the second medium risk, and the third high-risk patients. The three risk profiles for the validation data were created in the following way:

- low risk profile: 80% of the patients were sampled (without replacement) from the low-risk group, 50% from the medium-risk group, and 20% from the high-risk group;
- medium risk profile: 50% of the patients were sampled from the low-risk group, 50% from the medium-risk group, and 50% from the high-risk group;
- high risk profile: 20% of the patients were sampled from the low-risk group, 50% from the medium-risk group, and 80% from the high-risk group.

This sampling procedure was performed before generating each validation dataset and resulted in validation datasets that were half the size of the original datasets. In contrast, no sampling of patients was performed when generating the development datasets; all patients were used.

The prognostic indices were approximately normally distributed for all risk profiles and for both datasets. There was slight skewness, particularly in the low and medium risk profile datasets. For example, the (average) skewness in the HCM datasets was 0.8 (low risk profile), 0.4 (medium) and 0.1 (high). There was a similar trend in the breast cancer datasets, although the values were lower. The variance was largest for the medium profile datasets which was to be expected considering the sampling scheme. This suggests that the medium profile datasets contained more prognostic information than the low and high profile datasets. Finally, there was more prognostic information in the breast cancer data, as evidenced by the wider range of the corresponding prognostic indices.

#### Simulation results

**Fig. 2:**
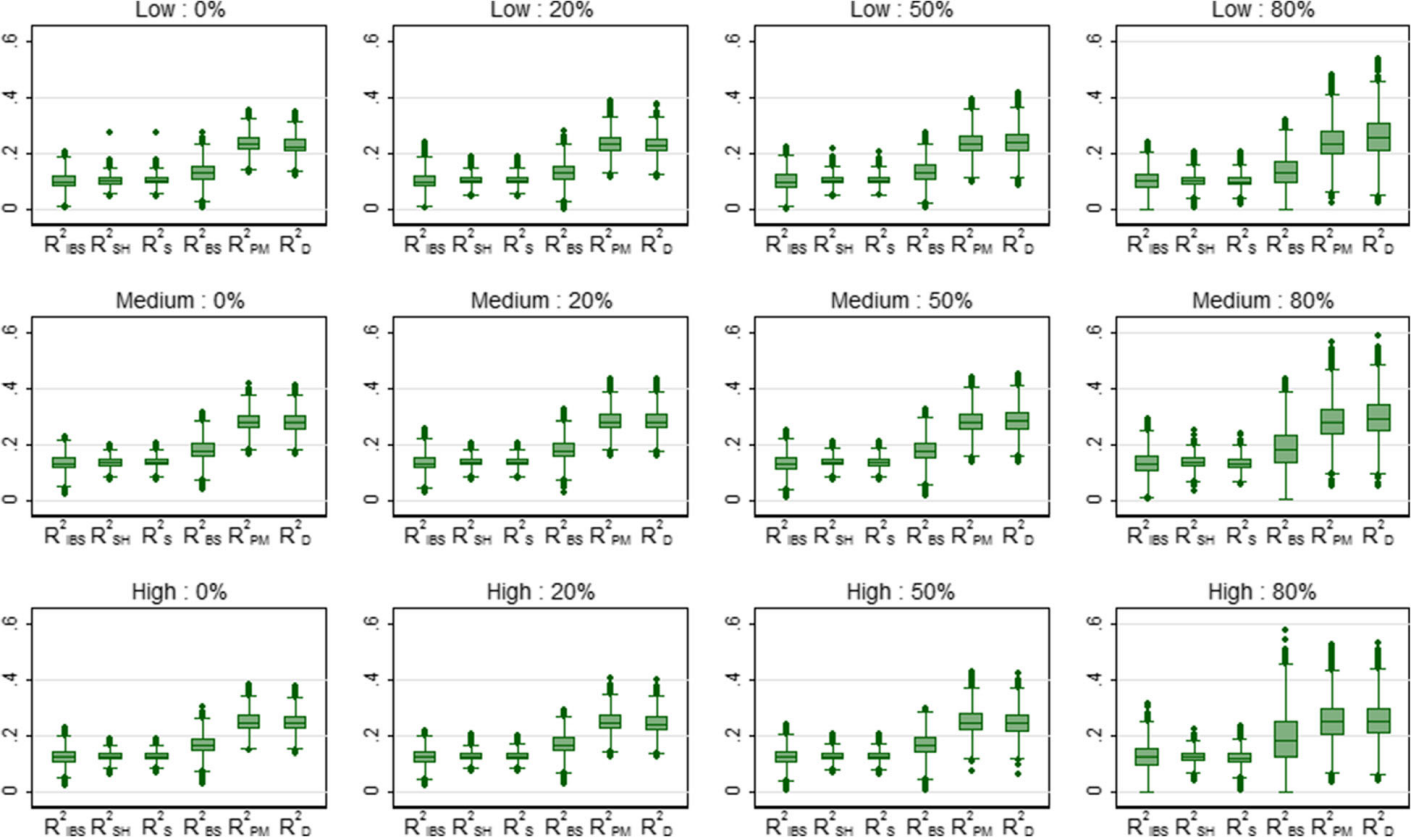
Box plots showing the distribution of the overall performance measures for 3 risk profiles (*low*, *medium* and *high*) and 4 levels of censoring (0, 20, 50 and 80%) for the breast cancer data over 5000 simulations

**Fig. 3:**
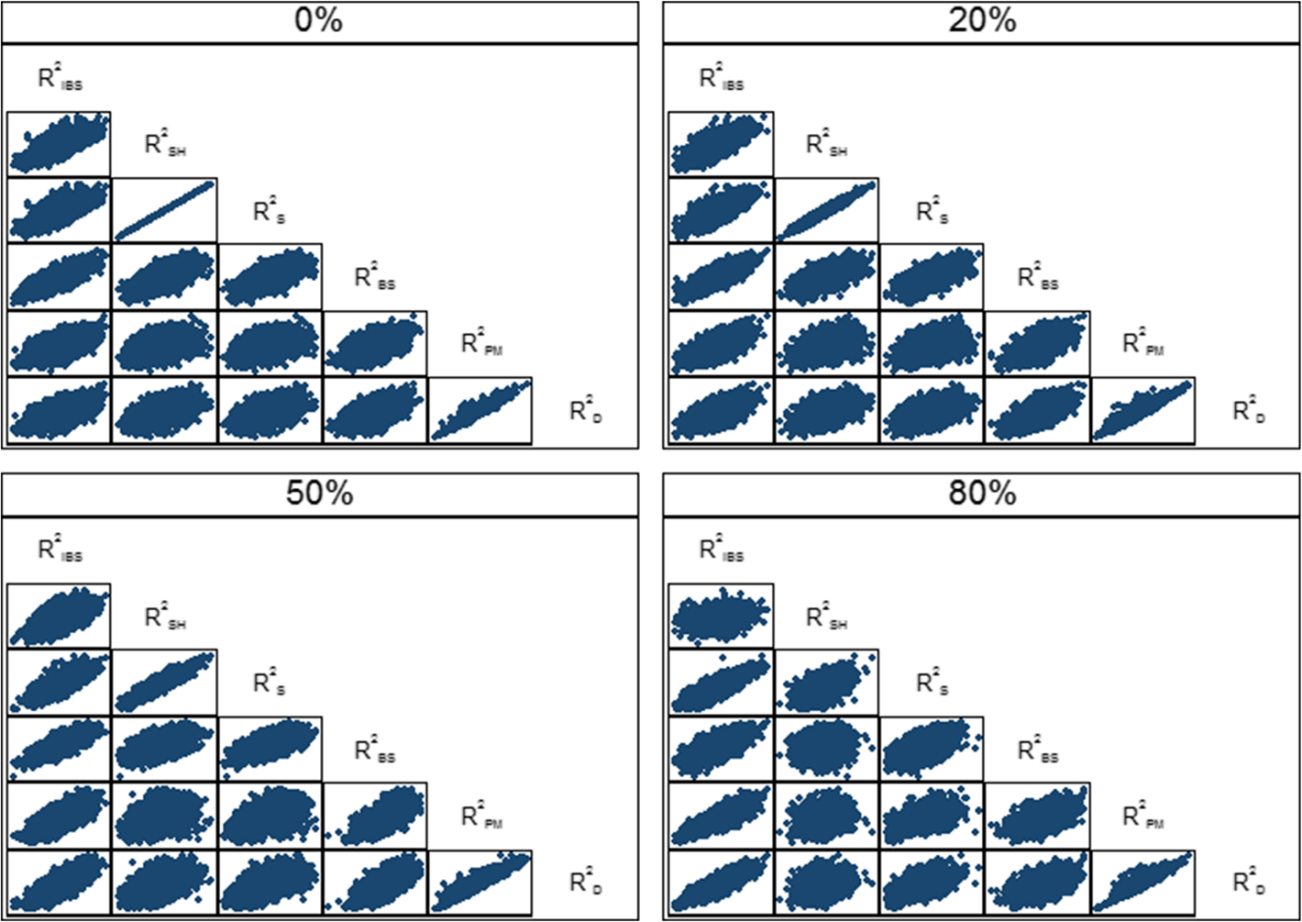
Scatter plot showing the relationships *between* the overall performance measures for the breast cancer data with the medium risk profile over 5000 simulations

Table 3 shows the mean values of the overall performance measures over 5000 simulations for the breast cancer data, for the four levels of censoring and three risk profiles. The three summary measures based on predictive accuracy (R^2^_IBS_, R^2^_SH_ and R^2^_S_) produced very similar values and were all unaffected by censoring. The values of these measures were highest for the medium risk profile simulations, where the patient characteristics were essentially the same in the development and validation samples, and lowest for the low risk profile simulations. Variability increased with increasing censoring, as expected, and was highest for R^2^_IBS_. This can clearly be seen in Fig. 2 which shows the distribution of the values of the performance measures over the 5000 simulations (a few negative values were deleted to aid clarity). R^2^_BS_, evaluated at 3 years, was also unaffected by censoring and achieved higher values in the medium risk profile simulations. R^2^_BS_ also produced some negative values (4%) when censoring was 80%. The two measures based on explained variation (R^2^_PM_ and R^2^_D_) produced similar values that were twice as large as the values obtained for R^2^_IBS_, R^2^_SH_ and R^2^_S_. R^2^_PM_ was unaffected by censoring but R^2^_D_ increased slightly as censoring increased. The relationships between the various overall performance measures are shown in Fig. 3 for the medium risk profile scenario. In particular, there was excellent agreement between the R^2^_SH_ and R^2^_S_ measures which weakened as censoring increased (ρ = 0.54 for 80% censoring). Also, there was good agreement between R^2^_PM_ and R^2^_D_ which seemed little affected by censoring (ρ = 0.95). Very similar relationships were seen for the low and high risk scenarios (results not shown).

**Table 3:** Mean (SD) of the overall performance measures for the breast cancer data over 5000 simulations

Profile	Censoring	R^2^_IBS_(3)	R^2^_SH_(3)	R^2^_S_(3)	R^2^_BS_(3)	R^2^_PM_	R^2^_D_
Low	0%	0.099 (0.032)	0.100 (0.018)	0.101 (0.018)	0.128 (0.037)	0.232 (0.034)	0.225 (0.034)
Low	20%	0.098 (0.033)	0.100 (0.019)	0.101 (0.019)	0.128 (0.038)	0.232 (0.038)	0.228 (0.038)
Low	50%	0.099 (0.034)	0.101 (0.019)	0.101 (0.019)	0.129 (0.040)	0.234 (0.045)	0.238 (0.048)
Low	80%	0.098 (0.041)	0.100 (0.024)	0.099 (0.023)	0.127 (0.060)	0.235 (0.065)	0.255 (0.075)
Medium	0%	0.131 (0.032)	0.133 (0.018)	0.135 (0.018)	0.176 (0.039)	0.279 (0.035)	0.277 (0.036)
Medium	20%	0.133 (0.032)	0.135 (0.018)	0.135 (0.018)	0.177 (0.040)	0.280 (0.038)	0.280 (0.038)
Medium	50%	0.131 (0.034)	0.135 (0.019)	0.134 (0.019)	0.176 (0.045)	0.279 (0.046)	0.283 (0.047)
Medium	80%	0.130 (0.045)	0.133 (0.025)	0.131 (0.025)	0.176 (0.082)	0.281 (0.068)	0.292 (0.071)
High	0%	0.121 (0.028)	0.123 (0.015)	0.125 (0.015)	0.165 (0.035)	0.247 (0.035)	0.243 (0.034)
High	20%	0.121 (0.028)	0.124 (0.016)	0.124 (0.016)	0.165 (0.038)	0.247 (0.038)	0.242 (0.037)
High	50%	0.121 (0.031)	0.125 (0.016)	0.124 (0.017)	0.164 (0.046)	0.247 (0.047)	0.243 (0.046)
High	80%	0.120 (0.048)	0.121 (0.022)	0.120 (0.026)	0.168 (0.114)	0.250 (0.070)	0.252 (0.071)

**Fig. 4:**
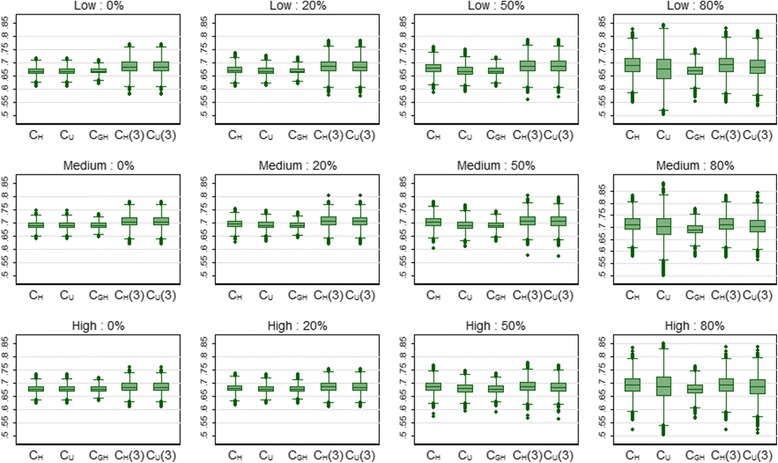
Box plots showing the distribution of the concordance measures for 3 risk profiles (low, medium and high) and 4 levels of censoring (0, 20, 50 and 80%) for the breast cancer data over 5000 simulations

**Fig. 5:**
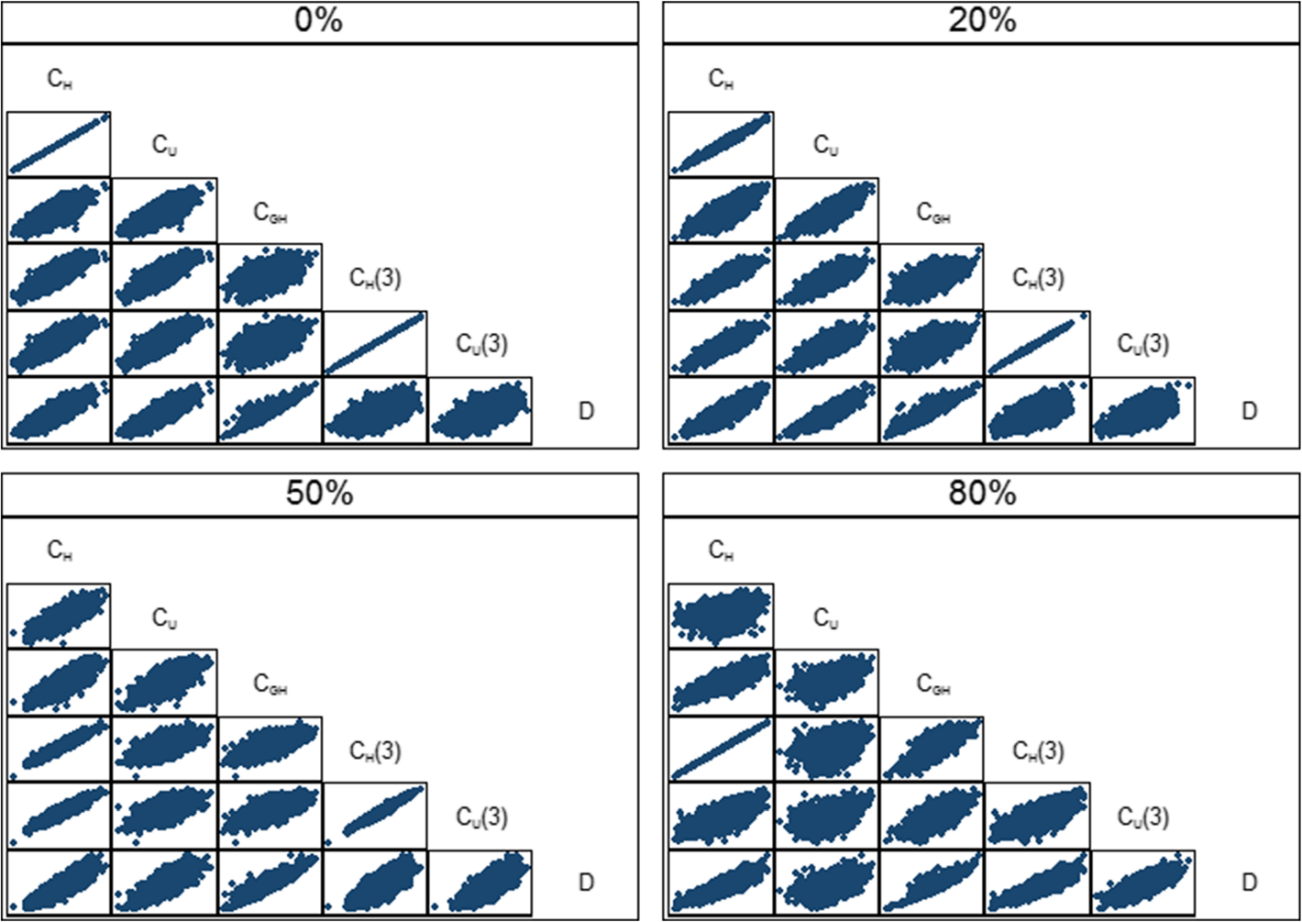
Scatter plot showing the relationships between the discrimination measures for the breast cancer data with the medium risk profile over 5000 simulations

Table 4 shows the mean values of the discrimination and calibration measures for the breast cancer data. The Harrell and Uno c-indices were estimated twice, first using all usable patient pairs (C_H_ and C_U_) and second by restricting the calculations by censoring times greater than 3 years (C_H_(3) and C_U_(3)). For 0% censoring, the C_H_ and C_GH_ mean values were very similar, and the C_H_ and C_U_ estimates were identical by definition. C_H_ tended to increase as censoring increased, whereas C_U_ and particularly C_GH_ were little affected. C_GH_ was the least variable of these three estimates. The variability in C_H_ and C_U_ was similar except for 80% censoring where the variability in C_U_ was far larger (Fig. 4). The increased variability was probably due to large values of the weights caused by the high degree of censoring. The mean value of C_H_(3) (and C_U_(3)) was slightly larger than that for C_H_ which suggests that the models were better able to discriminate within the first 3 years compared to across the whole follow-up period. Both C_H_(3) and C_U_(3) were relatively stable with respect to censoring, and the variability of both measures was similar. The calibration slope and particular the D statistic showed a slight tendency to increase with censoring. The relationships between the discrimination measures are shown in Fig. 5 for the medium risk profile scenario. In particularly, there was reasonable agreement between the concordance measures and D. The strong relationship between C_H_ and C_H_(3) for 80% censoring is explained by the fact that there were few observed failure times above 3 years with this level of censoring. Very similar relationships were seen for the low and high risk breast cancer scenarios (results not shown).

**Table 4:** Mean (SD) of the discrimination and calibration measures for the breast cancer data over 5000 simulations

Profile	Censoring	C_H_	C_U_(τ_max_)	C_GH_	C_H_(3)	C_U_(3)	D	Cal. Slope
Low	0%	0.667 (0.015)	0.667 (0.015)	0.667 (0.012)	0.684 (0.028)	0.684 (0.028)	1.103 (0.107)	0.981 (0.108)
Low	20%	0.670 (0.018)	0.667 (0.016)	0.667 (0.014)	0.684 (0.029)	0.684 (0.029)	1.111 (0.121)	0.982 (0.116)
Low	50%	0.679 (0.023)	0.668 (0.022)	0.668 (0.017)	0.687 (0.030)	0.685 (0.029)	1.144 (0.152)	0.987 (0.136)
Low	80%	0.689 (0.039)	0.673 (0.060)	0.667 (0.024)	0.690 (0.040)	0.684 (0.040)	1.197 (0.243)	0.989 (0.190)
Medium	0%	0.690 (0.015)	0.690 (0.015)	0.689 (0.013)	0.704 (0.023)	0.704 (0.023)	1.269 (0.113)	0.979 (0.101)
Medium	20%	0.694 (0.017)	0.690 (0.015)	0.690 (0.014)	0.705 (0.024)	0.704 (0.024)	1.278 (0.123)	0.984 (0.107)
Medium	50%	0.701 (0.022)	0.690 (0.021)	0.689 (0.017)	0.706 (0.026)	0.704 (0.026)	1.288 (0.152)	0.980 (0.126)
Medium	80%	0.711 (0.037)	0.698 (0.056)	0.689 (0.024)	0.711 (0.037)	0.704 (0.037)	1.316 (0.231)	0.986 (0.177)
High	0%	0.677 (0.015)	0.677 (0.015)	0.676 (0.013)	0.684 (0.021)	0.684 (0.021)	1.158 (0.108)	0.977 (0.108)
High	20%	0.679 (0.017)	0.677 (0.016)	0.676 (0.014)	0.684 (0.022)	0.683 (0.021)	1.155 (0.118)	0.979 (0.116)
High	50%	0.684 (0.023)	0.677 (0.021)	0.676 (0.018)	0.686 (0.025)	0.683 (0.024)	1.158 (0.148)	0.980 (0.139)
High	80%	0.692 (0.038)	0.683 (0.058)	0.676 (0.026)	0.692 (0.038)	0.685 (0.042)	1.187 (0.230)	0.987 (0.198)

**Fig. 6:**
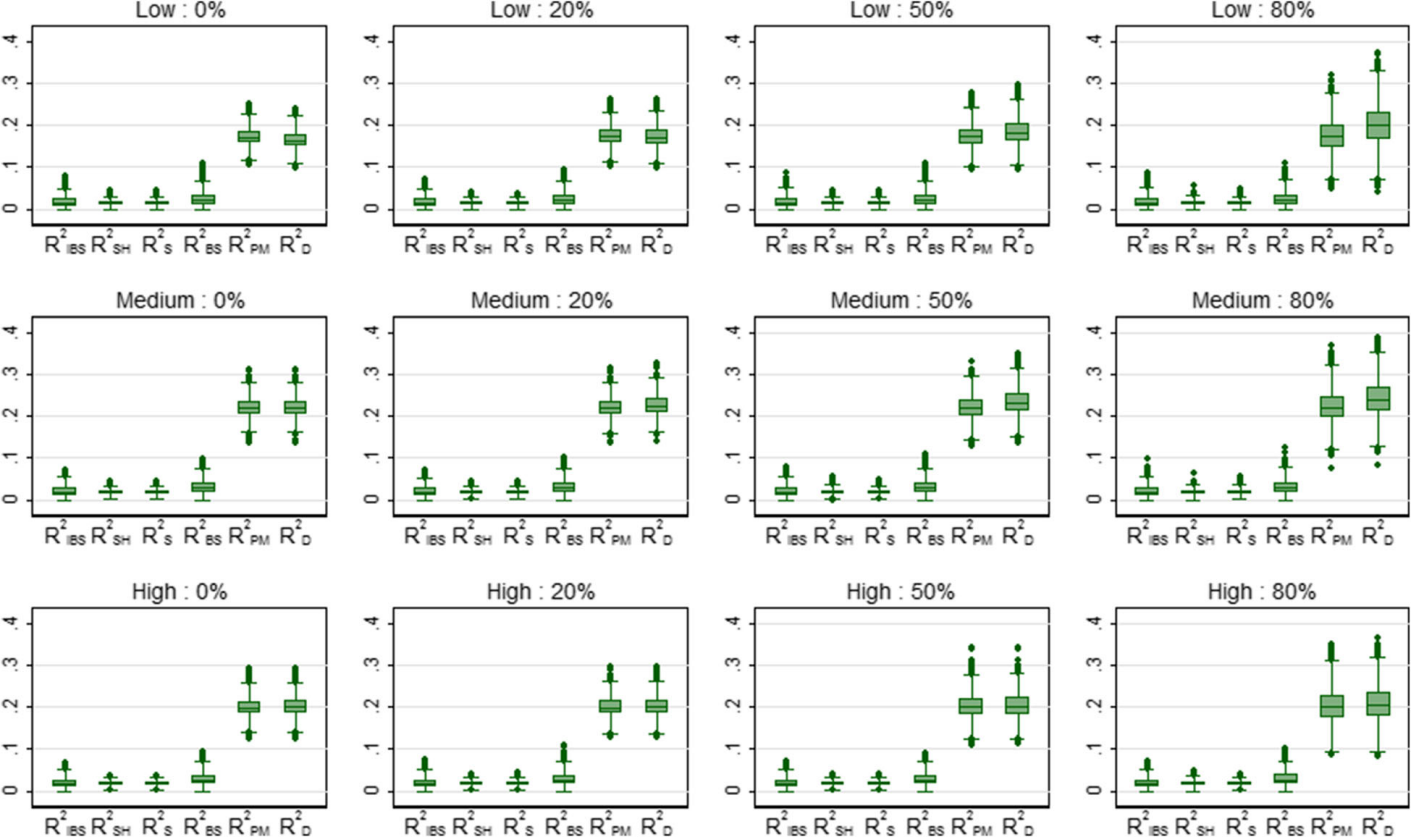
Box plots showing the distribution of the overall performance measures for 3 risk profiles (*low*, *medium* and *high*) and 4 levels of censoring (0, 20, 50and 80%) for the HCM data over 5000 simulations

The results for the overall performance measures for the HCM data can be seen in Table 5 and Fig. 6. The mean values were all lower than the corresponding values for the breast cancer data. In particular, the predictive accuracy values were considerably lower due to the relatively low number of events (5%) that occurred before 5 years. In addition there were many negative R^2^_IBS_ and R^2^_BS_ values (11 and 9% respectively). R^2^_D_ was affected by censoring in the low and medium risk profile simulations which may be explained by skewness in the prognostic index [6]. For example, the prognostic index was most skewed in the low risk profile HCM simulations, which is where greatest effect of censoring was observed. The relationships between the overall performance measures were similar, and often slightly stronger, than those seen in the breast cancer simulations (results not shown).

**Table 5:** Mean (SD) of the overall performance measures for the HCM data over 5000 simulations

Profile	Censoring	R^2^_IBS_(5)	R^2^_SH_ (5)	R^2^_S_ (5)	R^2^_BS_(5)	R^2^_PM_	R^2^_D_
Low	0%	0.013 (0.015)	0.013 (0.006)	0.014 (0.006)	0.020 (0.019)	0.173 (0.021)	0.166 (0.021)
Low	20%	0.013 (0.014)	0.013 (0.006)	0.013 (0.006)	0.020 (0.019)	0.173 (0.022)	0.173 (0.023)
Low	50%	0.014 (0.015)	0.013 (0.006)	0.013 (0.006)	0.020 (0.019)	0.174 (0.026)	0.184 (0.029)
Low	80%	0.014 (0.015)	0.014 (0.007)	0.014 (0.006)	0.020 (0.020)	0.174 (0.037)	0.201 (0.047)
Medium	0%	0.018 (0.014)	0.018 (0.006)	0.019 (0.006)	0.027 (0.019)	0.221 (0.022)	0.221 (0.023)
Medium	20%	0.018 (0.014)	0.018 (0.006)	0.019 (0.006)	0.027 (0.018)	0.221 (0.023)	0.226 (0.024)
Medium	50%	0.018 (0.014)	0.018 (0.006)	0.019 (0.006)	0.027 (0.019)	0.221 (0.028)	0.233 (0.031)
Medium	80%	0.018 (0.015)	0.018 (0.008)	0.019 (0.007)	0.027 (0.019)	0.222 (0.038)	0.241 (0.042)
High	0%	0.018 (0.013)	0.018 (0.005)	0.018 (0.005)	0.026 (0.017)	0.199 (0.022)	0.200 (0.022)
High	20%	0.018 (0.013)	0.018 (0.005)	0.018 (0.005)	0.027 (0.017)	0.199 (0.023)	0.201 (0.023)
High	50%	0.018 (0.013)	0.018 (0.006)	0.018 (0.005)	0.026 (0.017)	0.200 (0.028)	0.203 (0.029)
High	80%	0.018 (0.013)	0.018 (0.007)	0.018 (0.006)	0.026 (0.017)	0.201 (0.040)	0.206 (0.041)

**Fig. 7:**
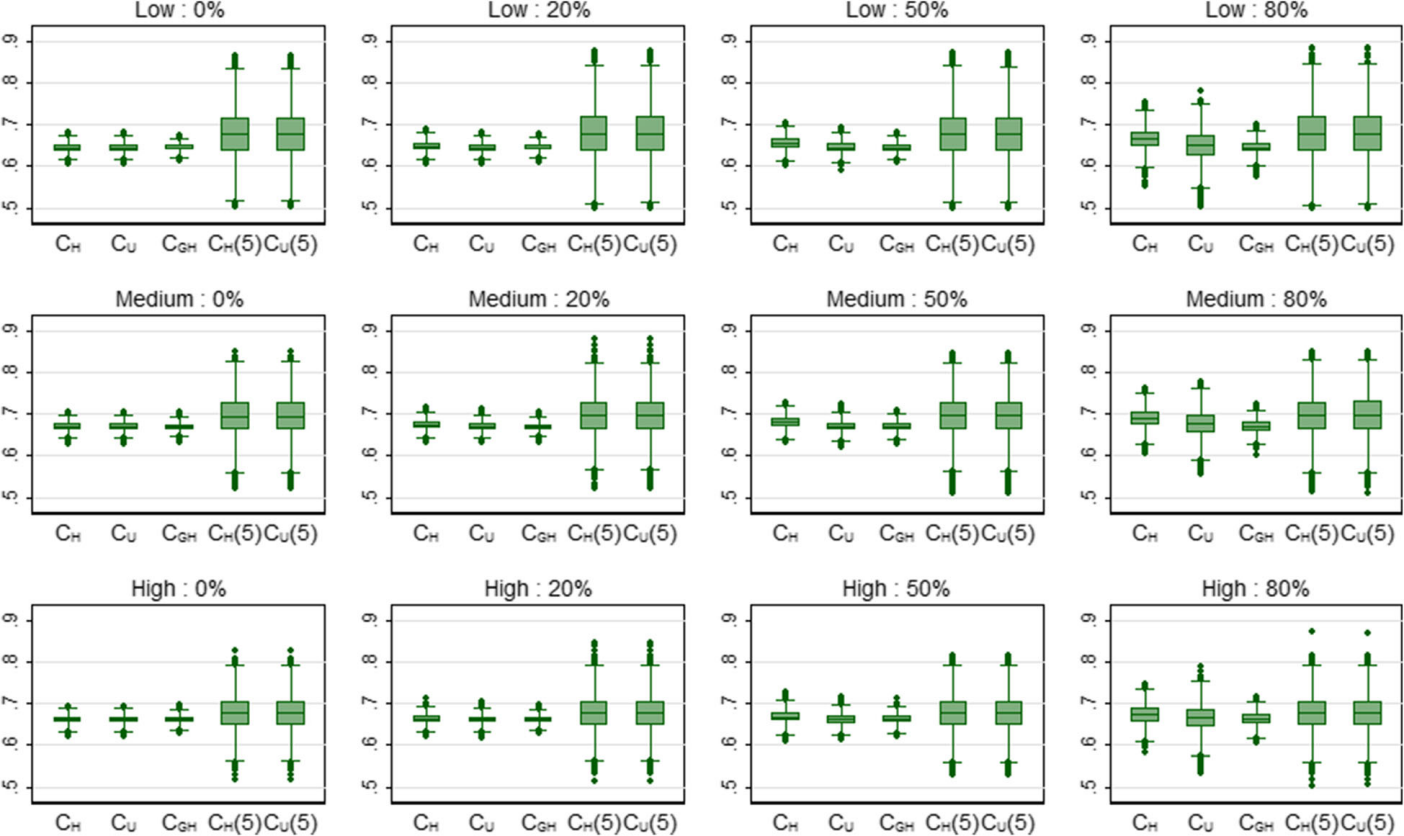
Box plots showing the distribution of the concordance measures for 3 risk profiles (*low*, *medium* and *high*) and 4 levels of censoring (0, 20, 50 and 80%) for the HCM data over 5000 simulations

The results for the discrimination and calibration measures for the HCM data can be seen in Table 6 and Fig. 7. As with the overall performance measures, the discrimination values were all lower than the corresponding values for the breast cancer data. C_H_ was again badly affected by censoring. In addition, D*,* like R^2^_D_, was also affected by censoring in the low and medium risk profile simulations. Also notable is the increased variability of the C_H_(5) and C_U_(5) measures compared to their unrestricted counterparts. This again is due to the relatively low number of events that occurred before 5 years and the consequent low number of patient pairs used to estimate both measures. Again, the relationships between the discrimination measures were similar to those seen in the breast cancer simulations (results not shown). In particular, there was excellent agreement between C_GH_ and D (*ρ* = 0.99).

**Table 6:** Mean (SD) of the discrimination and calibration measures for the HCM data over 5000 simulations

Profile	Censoring	C_H_	C_U_(τ_max_)	C_GH_	C_H_(5)	Cu(5)	D	Cal. Slope
Low	0%	0.645 (0.011)	0.645 (0.011)	0.645 (0.009)	0.675 (0.061)	0.675 (0.061)	0.911 (0.070)	0.983 (0.082)
Low	20%	0.649 (0.012)	0.645 (0.011)	0.645 (0.009)	0.676 (0.061)	0.676 (0.061)	0.934 (0.075)	0.986 (0.086)
Low	50%	0.656 (0.016)	0.645 (0.014)	0.645 (0.011)	0.676 (0.062)	0.676 (0.062)	0.971 (0.095)	0.989 (0.098)
Low	80%	0.666 (0.026)	0.649 (0.039)	0.645 (0.016)	0.676 (0.063)	0.676 (0.063)	1.025 (0.151)	0.988 (0.136)
Medium	0%	0.670 (0.010)	0.670 (0.010)	0.670 (0.009)	0.694 (0.049)	0.694 (0.049)	1.090 (0.072)	0.985 (0.075)
Medium	20%	0.674 (0.012)	0.670 (0.011)	0.670 (0.009)	0.695 (0.048)	0.695 (0.048)	1.105 (0.077)	0.986 (0.079)
Medium	50%	0.680 (0.015)	0.670 (0.013)	0.670 (0.011)	0.694 (0.049)	0.694 (0.049)	1.127 (0.097)	0.985 (0.091)
Medium	80%	0.688 (0.022)	0.675 (0.033)	0.670 (0.015)	0.695 (0.050)	0.695 (0.050)	1.153 (0.134)	0.989 (0.115)
High	0%	0.661 (0.011)	0.661 (0.011)	0.661 (0.009)	0.676 (0.043)	0.676 (0.043)	1.022 (0.070)	0.982 (0.079)
High	20%	0.663 (0.011)	0.661 (0.011)	0.661 (0.010)	0.677 (0.043)	0.677 (0.043)	1.025 (0.075)	0.983 (0.083)
High	50%	0.667 (0.015)	0.661 (0.013)	0.661 (0.011)	0.676 (0.043)	0.676 (0.043)	1.032 (0.092)	0.984 (0.097)
High	80%	0.672 (0.023)	0.664 (0.034)	0.661 (0.016)	0.676 (0.044)	0.676 (0.044)	1.042 (0.133)	0.987 (0.132)

## Discussion

The aim of this research was to review some of the promising performance measures for evaluating prediction models for survival outcomes, modify them if necessary for use with external validation data, and perform a simulation study based on two clinical datasets in order to make practical recommendations.

Measures based on predictive accuracy quantify the predictive ability of the prediction model, relative to a null model with no predictors, on a percentage scale and can be readily communicated to health researchers. The measures investigated in this study (R^2^_IBS_, R^2^_BS_, R^2^_SH_ and R^2^_S_) may be estimated for any survival prediction model provided that both the prognostic index and baseline survival function are available, although R^2^_SH_ also implicitly assumes that the model is correctly specified [12]. If the baseline survival function is not available, which is usually the case in practice [6], then one approach might be to estimate it using the validation data. This is a pragmatic choice as the baseline survival function is rarely presented in practice by model developers. An alternative, arguably better, approach would be to estimate the baseline survival function for the prediction model (with covariates), but not the null model, using the development data. This alternative approach was investigated in the case study and produced very similar results (not shown). A negligible difference in the baseline survival function is also reported in [6] when it was estimated using development and validation data separately. Similarly, for predictions from a null model, which are required for these measures, we suggest using the Kaplan-Meier estimate from the validation data.

Again for these measures, a choice of time-point is also required since the summary measures (R^2^_IBS_, R^2^_SH_ and R^2^_S_) are estimated over a specified range and R^2^_BS_ is estimated at a specified time-point. In practice, the choice of time-point will be guided by the clinical research question and the length of follow-up. For example, it is common for risk to be estimated at 5 years [4, 20]. The four predictive accuracy measures studied were not affected by censoring in the simulation study. In addition, the three summary measures (R^2^_IBS_, R^2^_SH_ and R^2^_S_) produced very similar values on average. However, the variability of R^2^_IBS_ was much greater than R^2^_SH_ and R^2^_S_ which suggests that use of the latter two measures might be preferred in practice if a summary measure is required. Hielscher et al. compared two of these measures, R^2^_IBS_ and R^2^_SH_, and had similar findings [12].

The measures based on explained variation (R^2^_PM_ and R^2^_D_) may be estimated for any proportional hazards model provided that the prognostic index is available, although we suggest that the prediction model is re-calibrated to the validation data before calculation of R^2^_PM_ to ensure that the survival times in the validation data are used in its calculation. Both measures provided very similar values in our simulations. R^2^_PM_ was robust to censoring, but R^2^_D_ tended to increase with censoring if the prognostic index was skewed.

Concordance measures are routinely used in practice since the concept of correctly ranking patient pairs can be readily communicated to health researchers [5]. C_H_ and C_U_ can be estimated for any survival prediction model that is able to rank patients. In addition, the calculation of C_U_ may also be restricted to a specified range of time, which may be useful to match with clinical aims or to compare concordance across different datasets. The calculation of C_H_ may also be restricted though it is not clear how often this is done in practice [37]. C_GH_ has a similar interpretation to the other concordance measures but requires that the model is correctly specified. As with R^2^_PM_, we suggest that the prediction model is re-calibrated to the validation data before calculation of C_GH_ to ensure that the survival times in the validation data are used. Harrell’s C_H_, in its unrestricted form, is probably the most used concordance measure in practice [5]. However, it was affected by censoring, which is a finding noted by others [16]. Specifically, C_H_ tended to increase for moderate to high levels of censoring, which is not an uncommon scenario with medical data, and is therefore likely to give an over-optimistic view of a prediction model’s discriminatory ability. Therefore, it cannot be recommended in such scenarios. In contrast, both C_U_ and C_GH_ were reasonably stable in the presence of censoring. C_GH_ was the less variable of the two measures as a consequence of it being model-based [16]. The restricted versions of C_H_ and C_U_ were little affected by censoring but care needs to be taken when selecting the time-point to ensure that the time period contains a reasonable number of events.

The remaining discrimination measure D has an appealing interpretation as it can be communicated as a (log) relative risk between low and high risk groups of patients. It requires that the proportional hazards assumption holds and that the prognostic index is normally distributed. As with R^2^_D_ it may be affected by censoring if the prognostic index is skewed [27]. The sole calibration measure under investigation, the calibration slope, was robust to censoring. It assumes that the proportional hazards assumption holds although more general approaches are described by van Houwelingen [33, 38].

## Conclusions

Harrell’s C_H_ is routinely reported to assess discrimination when survival prediction models are validated [5]. However, based on our simulation results, we recommend that C_U_ is used instead to quantify concordance when there are moderate levels of censoring. Alternatively, C_GH_ could be considered, especially if censoring is very high, but we suggest that the prediction model is re-calibrated first. The restricted version of C_H_ may also be used provided that the time-point is chosen carefully. We also recommend that D is routinely reported to assess discrimination since it has an appealing interpretation, although the distribution of the prognostic index would need to be checked for normality. ‘Overall performance’ measures are perhaps under used in practice. Our recommendation would be to use any of the predictive accuracy measures and provide the corresponding predictive accuracy curves. In particular, R^2^_SH_ and R^2^_S_ have relatively low variability. The calibration slope is a useful measure of calibration and recommended to report routinely. In addition, one could investigate calibration graphically, for example by comparing observed and predicted survival curves for groups of patients [6]. Finally, we also recommend to investigate the characteristics of the validation data such as the level of censoring and the distribution of the prognostic index derived in the validation setting before choosing the performance measures.

## Abbreviations

FP: Fractional Polynomial
HCM: Hypertrophic Cardiomyopathy
